# Association of CDKN2A overexpression with poor prognosis, radioresistance, and immunosuppression in colorectal cancer

**DOI:** 10.3389/fcell.2026.1955910

**Published:** 2026-10-16

**Authors:** Yaobin Lin, Zhihong Wang, Gaoda Ju, Yueying Zhang, Zongwei Huang, Shan Liu, Benhua Xu, Zhao Zhan

**Affiliations:** 1 Department of Radiation Oncology, Fujian Medical University Union Hospital, Fujian Key Laboratory of Intelligent Imaging and Precision Radiotherapy for Tumors (Fujian Medical University), Clinical Research Center for Radiology and Radiotherapy of Fujian Province (Digestive, Hematological and Breast Malignancies), Fuzhou, Fujian, China; 2 Department of Hematology, Fuzhou University Affiliated Provincial Hospital, Shengli Clinical Medical College of Fujian Medical University, Fujian Provincial Hospital, Fuzhou, Fujian, China; 3 Department of Radiation Oncology, Clinical Oncology School of Fujian Medical University, Fujian Cancer Hospital, Fuzhou, Fujian, China; 4 Fujian Children’s Hospital, Fujian Medical University, Fuzhou, Fujian, China; 5 Department of Laboratory Medicine, Zhongnan Hospital of Wuhan University, Wuhan, Hubei, China

**Keywords:** CDKN2A, colorectal cancer, immune microenvironment, prognosis, radiotherapy

## Abstract

**Background:**

In colorectal cancer (CRC), dysregulated cyclin-dependent kinase inhibitor 2A (CDKN2A) contributes to disease progression. This investigation sought to explore the link between *CDKN2A* expression and the effectiveness of radiotherapy in CRC, and to investigate the underlying mechanisms.

**Methods:**

Transcriptomic and clinical datasets were procured from the Cancer Genome Atlas and Gene Expression Omnibus databases. Immune cell infiltration was assessed using xCell, CIBERSORT, and EPIC methods. Biological mechanisms were investigated through the utilization of Gene Ontology and Kyoto Encyclopedia of Genes and Genomes. HCT 116 cells were used for *in vitro* and *in vivo* experiments.

**Results:**

Bulk and single-cell transcriptome analyses and immunohistochemistry consistently revealed elevated *CDKN2A* expression in tumor specimens from patients with CRC. Patients with tumors that were nonresponsive to neoadjuvant chemoradiotherapy had significantly higher *CDKN2A* expression than those of responders (*P* = 0.039). *CDKN2A* knockdown in tumor led to a marked decrease in the proliferation, invasion, and migration capabilities, and this effect was enhanced by combined X-ray irradiation. Elevated *CDKN2A* expression was linked to markedly worse survival. In the tumor immune microenvironment, *CDKN2A* expression was negatively linked to CD8^+^ cell infiltration and positively correlated with the levels of immunosuppressive factors. Functional enrichment analysis indicated that elevated *CDKN2A* expression is linked to transcriptional regulation, amino acid metabolism, protein folding, and the TGF-β signaling pathway.

**Conclusion:**

In CRC, *CDKN2A* overexpression is associated with unfavorable responses to radiotherapy, an immunosuppressive tumor microenvironment, and poor prognosis. Suppression of *CDKN2A* expression could potentially improve therapeutic outcomes in CRC patients. More experimental and clinical studies are still needed to verify and further distinguish the similarities and differences between colon cancer and rectal cancer.

## Background

Colorectal cancer (CRC) stands as the second most prevalent cause of cancer-related mortality globally and ranks as the third most frequently diagnosed malignancy (Siegel et al., 2025). The annual incidence and mortality rate of CRC is 35.1 and 12.9 per 100,000 population, respectively. The mortality rate is gradually increasing (Shen et al., 2025), particularly in younger adults. In addition, 5%–30% of patients experience recurrence after tumor resection (Chakrabarti et al., 2020; Ahmed, 2020). Therefore, CRC is a major global health concern. The initial treatment for CRC can include endoscopic removal, colectomy, chemotherapy, and radiotherapy. However, few studies are available on the use of radiotherapy for colon cancer are available; and the pathological complete response (pCR) rate after neoadjuvant chemoradiotherapy for rectal cancer is unsatisfactory.

The high mobility of the colon and sensitivity of the small intestine and other organs to irradiation restrict the use of radiotherapy in colon cancer. According to the National Comprehensive Cancer Network guidelines, radiotherapy should be regarded as a conversion treatment for unresectable or medically inoperable non-metastatic stage T4 colon cancers. A study of 15,207 patients with stage T4 colon cancer reported that compared with patients not receiving neoadjuvant radiotherapy, patients receiving neoadjuvant radiotherapy had an improved R0 resection rate (87.2% vs. 79.8%, *P* = 0.009) and 5-year overall survival (OS) (62.0% vs. 45.7%, *P* < 0.001) (Hawkins et al., 2019). However, other studies have reported that radiotherapy has no benefit in patients with locally advanced colon cancer (Zhou et al., 2019; Krishnamurty et al., 2018). Therefore, enhancing radiosensitivity is crucial for converting unresectable colon cancer into resectable colon cancer.

For rectal cancer, the pCR rate after neoadjuvant chemoradiotherapy is 15%–30% (Erlandsson et al., 2017; Schmoll et al., 2012; Bahadoer et al., 2021; Zhu et al., 2020). Multiple investigations have shown that incorporating immunotherapy into chemoradiotherapy during the neoadjuvant treatment phase can boost the pCR rate to over 40% (Yang et al., 2024; Wang et al., 2025). This is because radiotherapy can enhance the effectiveness of immunotherapy. Radiotherapy can induce immunogenic death in tumor cells, releasing tumor antigens, promoting the infiltration of CD8^+^ T-cells and dendritic cells, while eliminating regulatory T-cells (Tregs) and M2-type macrophages, thereby alleviating the immunosuppressive microenvironment (Zain and Hanona, 2021; Zing et al., 2018). Therefore, identifying molecules that can not only improve tumor radiosensitivity but also enhance antitumor immunotherapy could potentially transform clinical management.

Cyclin-dependent kinase inhibitor 2A (CDKN2A) can maintain cell arrest in the G1 phase by inhibiting CDK4/6 kinase activity and can also initiate DNA repair or apoptosis by activating p53 (Parkinson et al., 1997). *CDKN2A* deletion may change the cell cycle distribution, inhibit the DNA damage-repair pathway, and affect radiosensitivity. *CDKN2A* expression is linked to PD-1 and PD-L1 expression in cervical and bladder urothelial cancer (Song et al., 2025). This investigation sought to study the relationship between *CDKN2A* and radiosensitivity and immunity in CRC using bulk transcriptomics, single-cell transcriptomics, clinical pathology and experimental research, and to explore potential mechanisms whereby *CDKN2A* influences the response to radiotherapy and immunotherapy.

## Methods

### Data extraction

Gene expression and clinical information of CRC patients were procured from The Cancer Genome Atlas (TCGA) (https://portal.gdc.cancer.gov/) and Gene Expression Omnibus (GEO) (https://www.ncbi.nlm.nih.gov/gds/) databases. Details regarding the datasets utilized in this investigation are depicted in Supplementary Table S1. The Robust Multichip Average algorithm was applied for data normalization (Irizarry et al., 2003). Human radiation response-related genes (RTRRGs) were obtained from Hou et al. (2014) (Supplementary Table S2). The study identified 688 RTRRGs by integrating five GEO datasets (GSE9712, GSE9713, GSE9714, GSE48503, and GSE20549), which compared the gene expression profiles of radiosensitive and radioresistant cancer cells in different cancer types. Differentially expressed genes (DEGs) were identified using a fold-change >1.5 and a false discovery rate (FDR) ≤0.05 as the cutoff criteria.

Single-cell transcriptome sequencing data were analyzed using the TISCH2 platform (http://tisch.compbio.cn/). Immunohistochemical data were procured from the Human Protein Atlas (https://www.proteinatlas.org/) and gene copy number variation (CNV) data were sourced from UCSC Xena (http://xena.ucsc.edu/). Drug sensitivity screening was conducted using the Genomics of Drug Sensitivity in Cancer (GDSC), Cancer Therapeutics Response Portal (CTRP), and Profiling Relative Inhibition Simultaneously in Mixtures (PRISM) databases. The research flow of this study is shown in Figure 1.

**FIGURE 1:**
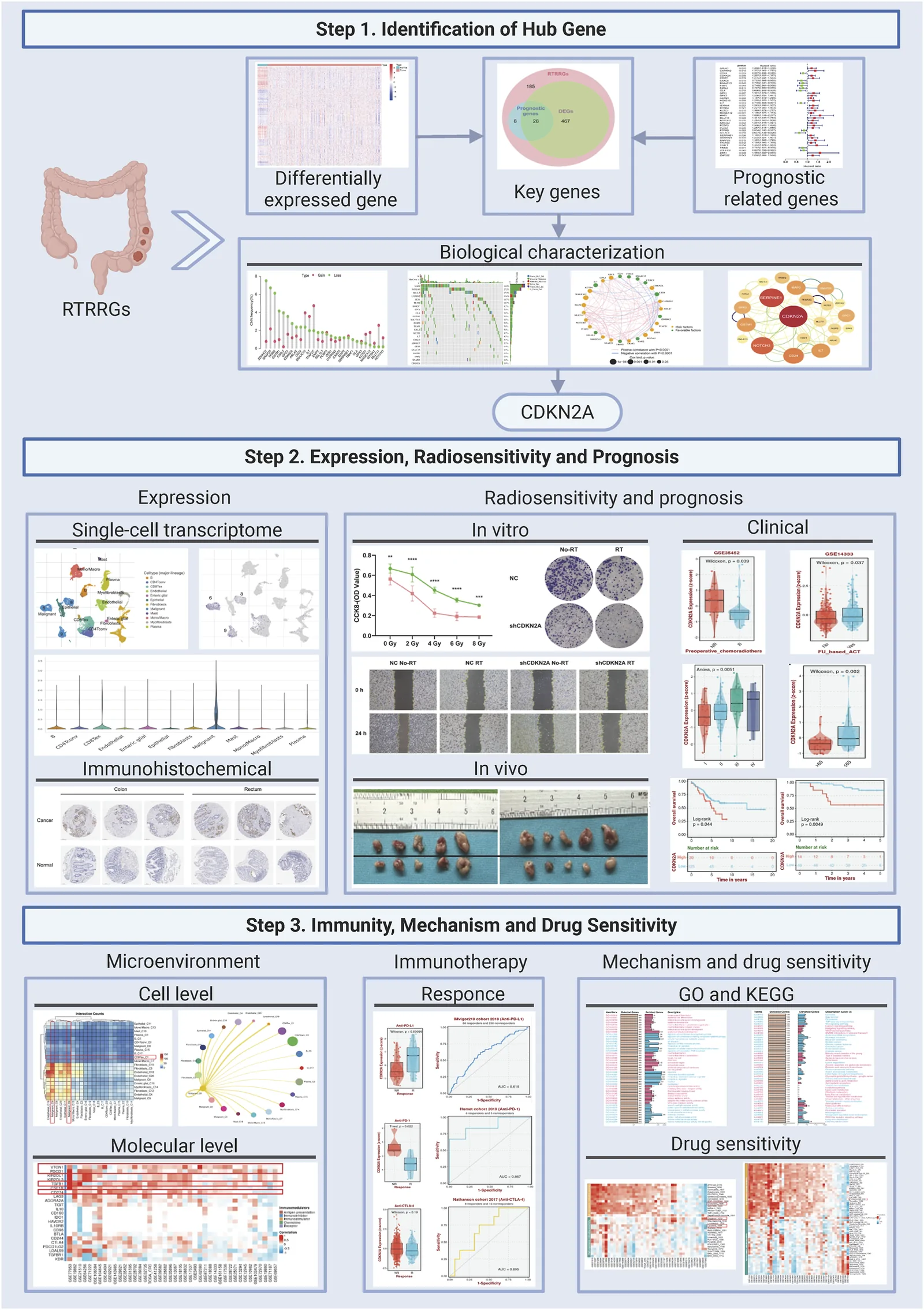
The research flowchart of this study. RTRRGs, radiation response-related genes; NC, negative control; No-RT, no-radiation; RT, radiation; GO, Gene Ontology; KEGG, Kyoto Encyclopedia of Genes and Genomes.

### Identification of RTRRGs related to survival

The RTRRG expression levels in tumor and normal tissues were compared to identify DEGs using the R software package “limma,” with the FDR set at <0.05. RTRRGs significantly associated with OS were identified through univariate Cox regression analysis, with a statistical threshold of *P* < 0.05. The R package “RCircos” was utilized to generate CNV maps depicting the distribution of 28 RTRRGs across 23 chromosome pairs. A waterfall plot of somatic tumor mutations was prepared using the Sangerbox platform (http://vip.sangerbox.com). The STRING (https://cn.string-db.org/) database was utilized for constructing a protein-protein interaction (PPI) network, and visualization was performed using Cytoscape software (v. 3.8.2).

### Tumor immune microenvironment and functional enrichment analysis

The relative abundance of different immune cells and immunomodulators was measured using the xCell, CIBERSORT, and EPIC methods. Immunotherapy information was procured from The Cancer Immunome Atlas (TCIA) (https://tcia.at). Biological functions and pathways linked to diminished and elevated CDKN2A expression levels were determined through the Gene Ontology (GO) and Kyoto Encyclopedia of Genes and Genomes (KEGG) databases (Yu et al., 2012). Enriched genes were defined according to the threshold values of *P* < 0.05 and FDR <25%.

### Cell culture and treatment

The human CRC cell line HCT 116 was procured from Fujian Medical University (Fuzhou, China). HCT 116 cells underwent cultivation in Dulbecco’s Modified Eagle Medium (DMEM) (Shanghai Basalmedia Technologies, China) comprising 10% fetal bovine serum (FBS) (PAN-Biotech GmbH, Aidenbach, Germany) within a 5% CO_2_ incubator maintained at 37 °C. Short hairpin RNA (shRNA) targeting *CDKN2A* were synthesized by Fujian Anburui Biotechnology Co., Ltd. (Fuzhou, China). The shRNA sequences utilized are depicted in Supplementary Table S3.

### *In-vivo* experiments

Female BALB/c mice (6–8 weeks old) were purchased from the Shanghai Wushi Experimental Animal Center (Shanghai, China). To construct the tumor-bearing model, 2 × 10^6^ NC cells or 2.5 × 10^6^ shCDKN2A cells were injected subcutaneously into the armpit and hindlimb. On the seventh day after inoculation, intervention was started. When the tumor volume reached 1000 mm^3^ (tumor volume = length × wide × wide × 0.5), the animal feeding was ended. Tumor inhibition rate = (1 - tumor volume of RT group/tumor volume of No-RT group) × 100%. All animal studies were approved by the Animal Care Committee of Fujian Medical University.

### X-ray irradiation

HCT 116 cells were exposed to perpendicular beam radiation utilizing a medical linear accelerator. The radiation doses administered were 0, 2, 4, 6, and 8 Gy, applied at a dose rate of 400 MU/min with 6 MeV energy, and incorporating 2 cm tissue-equivalent material as surface filler during the radiation procedure.

The subcutaneous tumor was irradiated by biological X-ray irradiator (Rad Source Technologies, USA). The irradiation condition is 225 kV, 17.7 mA, 0.3 mm copper alloy filter, no reflector is used, the irradiation dose is 9 Gy, and the dose rate is 1.634 Gy/min.

### Reverse-transcription PCR

RNA extraction from experimental samples was conducted utilizing the TRIzol extraction protocol. Subsequently, first-strand cDNA synthesis was carried out employing a commercial reverse-transcription kit (Yeasen Biotechnology Co. Ltd., Shanghai, China). Total RNA served as the initial template for this reaction. The details of reverse-transcription PCR (RT-PCR) procedure are shown in the Supplementary methods section.

### Western blot

A protease inhibitor was incorporated into the radioimmunoprecipitation assay (RIPA) buffer. Protein quantification was performed using the BCA protein assay kit. After electrophoresis and antibody incubation, the protein expression was visualized by chemiluminescence detection. The details of procedure are shown in the Supplementary methods section.

### Cell viability assessment

Cell viability was assessed utilizing the Cell Counting Kit-8 (CCK-8) viability assay (MedChemExpress, Princeton, NJ, USA). Absorbance at 450 nm was detected using a microplate reader. The details of procedure are shown in the Supplementary methods section.

### Clonogenic assay

Cells were digested using trypsin and seeded into 6-well plates at 1,000 cells per well, then incubated overnight. After irradiation treatment, cells were maintained in culture for 10–14 days and stained using crystal violet. The cellular survival rate was determined using the following formula: Cell survival rate = (clonogenic rate of irradiated cells/clonogenic rate of control cells) × 100%.

### Wound-healing and transwell assays

Cells were maintained at 37 °C until reaching 90% confluency and scratches were generated using 200-μL pipette tips. Detached cellular debris was eliminated through phosphate-buffered saline washing, followed by cell irradiation. Wound healing progression was monitored at 0- and 24-h post-scratch using an inverted microscope.

After irradiation, cells underwent dilution in serum-free medium before being placed into the upper chamber. Medium containing 10% FBS was added to the lower chamber as a chemoattractant. After 48 h of incubation, cells that had migrated to the lower surface underwent fixation and staining. The details of procedure are shown in the Supplementary methods section.

### Immunohistochemistry

The paraffin was frozen and sliced. After a series of processing according to the procedure, the slices were sealed with neutral gum. The details of procedure are shown in the Supplementary methods section. Tumor necrosis rate = necrotic area/total tissue area × 100%.

### Statistical analysis

Statistical analyses were conducted utilizing R software (v 3.6.3) (https://www.r-project.org/). DEGs between cancer and normal samples were evaluated through the Wilcoxon signed-rank test. Survival assessment was performed using Kaplan–Meier curves. The χ^2^ test was employed to establish the statistical significance of categorical variable differences between groups. Associations between continuous variables were evaluated using Spearman rank correlation coefficients. The paired-sample T-test was utilized to evaluate the significance of differences between paired samples. *P* values below 0.05 were deemed statistically significant.

## Results

### Screening RTRRGs related to prognosis of patients with CRC

First, 495 genes with differential expression between tumor and normal tissues (Figure 2A; Supplementary Table S4) and 36 genes significantly related to OS were identified from the 688 RTRRGs (Figure 2B). Through the intersection of DEGs and prognosis-related genes, 28 genes were identified as the key CRC genes (Figure 2C). The expression of these 28 key genes in tumor and adjacent tissues was displayed through a heatmap (Figure 2D). In addition, the CNVs, chromosome position, and gene mutations of these 28 genes were assessed (Figures 3A–C). Fourteen of the 28 genes had decreased copy numbers. However, the copy numbers of *MAP2*, *TRAF1*, and *GLA* genes increased or decreased at the same frequency. The three genes with the highest mutation frequencies were *MAP2* (35.0%), *NOTCH3* (27.2%), and *SEL1L3* (17.5%). The mutation rate of *CDKN2A* was 1%. The gene co-expression network and PPI are shown in Figures 3D,E, respectively. Since *CDKN2A* exhibits the most extensive interactions with other prognosis-related RTRRGs in the PPI network, it was selected as the hub gene for further investigation.

**FIGURE 2:**
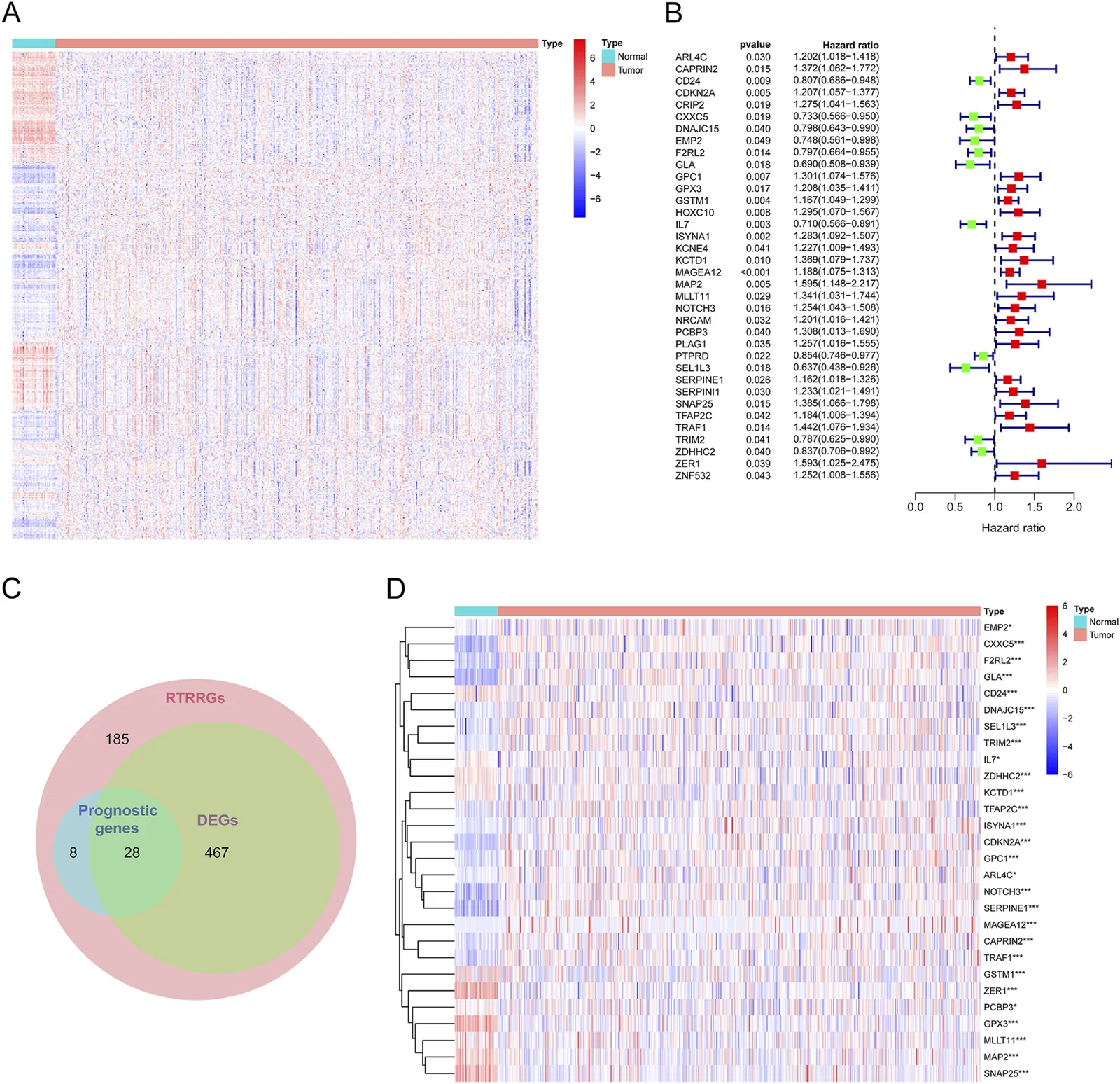
Screening the RTRRGs associated with the prognosis of colorectal cancer. **(A)** Heatmap of RTRRGs that are differentially expressed in cancer and adjacent tissues. **(B)** Forest plot of RTRRGs associated with prognosis based on univariate Cox analysis. **(C)** The relationship between differentially expressed genes in cancer and adjacent tissues and prognostic-related genes. **(D)** Heatmap of the expression levels of 28 key genes in cancer and adjacent tissues. RTRRGs, radiation response-related genes. *p < 0.05; **p < 0.01; ***p < 0.001.

**FIGURE 3:**
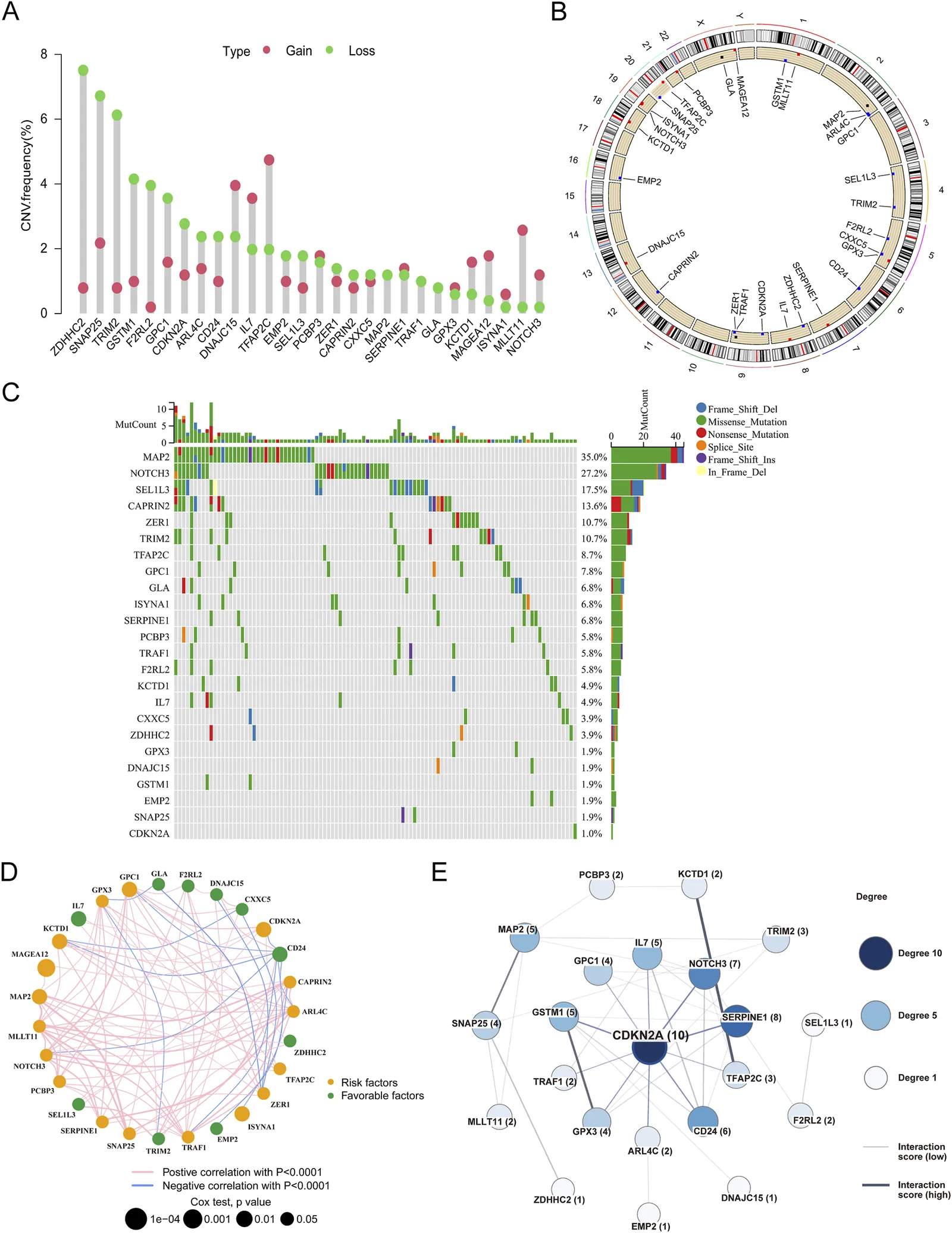
Biological characterization of the 28 RTRRGs in colorectal cancer. **(A)** The CNV frequency of RTRRGs. The height of the column represents the alteration frequency. **(B)** The location of CNV alteration of RTRRGs on 23 chromosomes. The blue dot represents the deletion frequency and the red dot represents the amplification frequency. **(C)** The mutation frequency of RTRRGs in patients with colorectal cancer. Each column represents an individual patient. **(D)** Interaction among the 28 RTRRGs. The size of the circle represents the effect of each gene on prognosis. The lines linking genes represent their interactions, and thickness shows the correlation strength between genes. **(E)** PPI network related to RTRRGs. The darker the blue color is, the more proteins it interacts with. The numbers within parentheses represent the quantities of proteins that interact with them. The lines linking the proteins represent their interactions and thickness shows the correlation strength. RTRRGs, radiation response-related genes; CNV, copy number variation; PPI, protein-protein interaction.

### Single-cell transcriptomics and immunohistochemistry proved that CDKN2A was highly expressed in CRC cells

The distribution of *CDKN2A* across various cell subsets was examined through single-cell transcriptomic analysis. Twenty-1 cell clusters, comprising 12 cell types, were identified in CRC tissue (Figures 4A,B; Supplementary Figure S1). Among the cells, three subtypes of tumor cells were identified, suggesting that CRC cells are heterogeneous. *CDKN2A* was highly expressed in the C6 and C9 subtypes (Figures 4C,D). In addition, we measured the *CDKN2A* protein level in CRC cells using immunohistochemistry. The results confirmed that *CDKN2A* was upregulated in CRC samples compared with normal tissues (Figure 4E).

**FIGURE 4:**
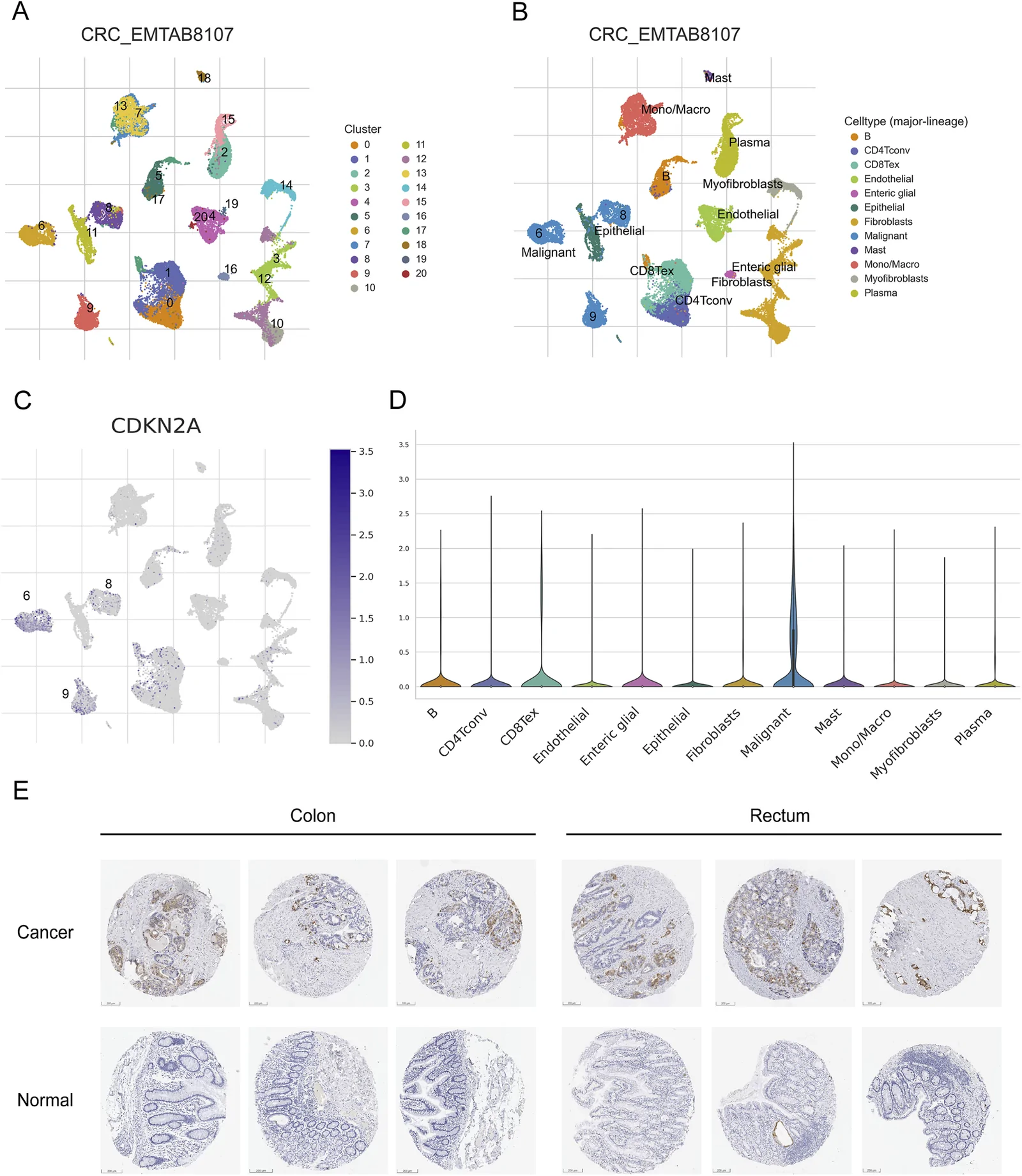
The expression of CDKN2A in colorectal cancer. UMAP of the distribution of **(A)** 21 cell clusters and **(B)** 12 cell types identified from single-cell transcriptomic dataset. The expression of CDKN2A in **(C)** 21 cell clusters and in **(D)** 12 types of cells. **(E)** Immunohistochemical detection of the expression of CDKN2A in tumor tissues and normal tissues of colorectal cancer.

### Relationship between CDKN2A and radiotherapy sensitivity

Tumor samples were obtained from 46 patients with rectal cancer prior to treatment. These patients all subsequently received neoadjuvant long-term radiotherapy and chemotherapy (radiation dose 50.4 Gy/28F and fluorouracil and leucovorin calcium [UTF/LV] chemotherapy) and underwent standardized surgery. The patients were split into two groups according to whether they responded to neoadjuvant therapy. *CDKN2A* expression was higher in tumor tissues of patients who did not respond to radiotherapy and chemotherapy than in those who responded (*P* = 0.039; Figure 5A). We than reviewed the postoperative pathology and follow-up data of 290 patients with rectal cancer who underwent surgery and 5-fluorouracil (5-FU)-based adjuvant chemotherapy/radiotherapy (radiation dose, 50.4Gy/28F). The *CDKN2A* expression in tumor tissues from patients experiencing recurrence were elevated versus those from patients who did not experience recurrence (*P* = 0.037; Figure 5B).

**FIGURE 5:**
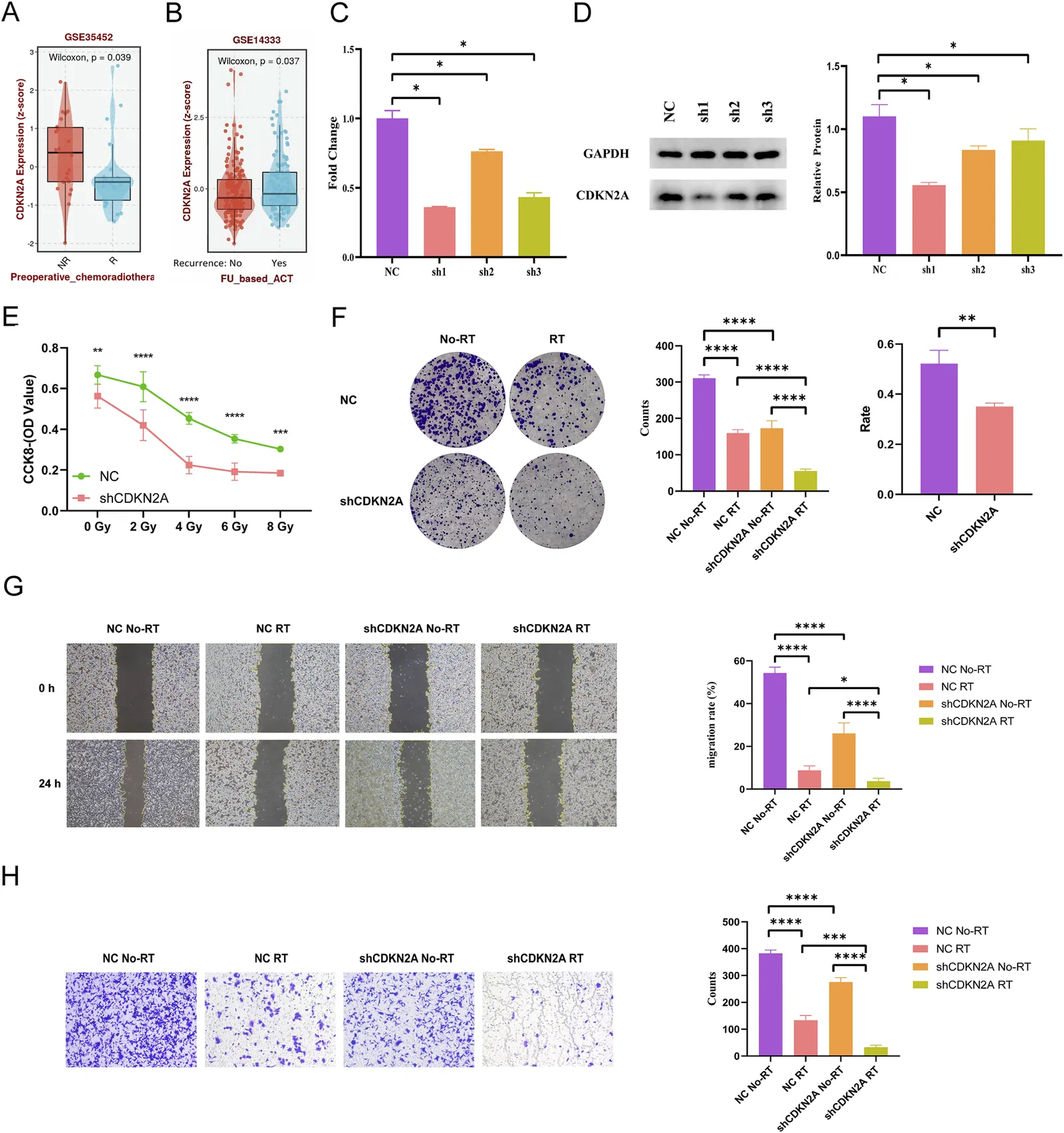
The relationship between CDKN2A and radiosensitivity. **(A)** Box plot of CDKN2A expression in different treatment response groups of patients after preoperative chemoradiotherapy. **(B)** Box plot of CDKN2A expression in the recurrent and non-recurrent populations. All the patients underwent surgery and 5-fluorouracil-based adjuvant chemotherapy/radiotherapy. **(C)** Bar chart showing the gene expression levels of CDKN2A in different intervention groups. Use shRNA to knock down the CDKN2A gene in the cells. **(D)** The Western blot showing the protein expression levels of CDKN2A in different intervention groups. The protein content is calculated as the relative value based on the color intensity of the protein bands in the gel. The statistical results are presented in a bar chart. **(E)** Line graph showing the vitality of cells after being exposed to different radiation doses. The pink line represents the negative control group, and the green line represents the sh*CDKN2A* group. **(F)** Cloning formation diagrams of different intervention groups. The graphs of the **(G)** wound-healing and **(H)** transwell results for different intervention groups. NR, no-response; R, response; NC, negative control; No-RT, no-radiation; RT, radiation. *p < 0.05; **p < 0.01; ***p < 0.001; ****p < 0.0001.

shRNA was utilized to knock down *CDKN2A* expression in HCT 116 cells to elucidate the impact of CKDN2A on the radiosensitivity of CRC. The RT-PCR results showed that *CDKN2A* mRNA expression in the shRNA treatment (sh1, sh2, and sh3) groups was markedly diminished versus the negative control (NC) group (Figure 5C). The effect of knockdown was most marked in the sh1 group, and this was confirmed by Western blot (Figure 5D; Supplementary Figure S2). Therefore, the sh1 group was selected for subsequent experiments. The CCK-8 assay demonstrated that relative to the NC group, the sh*CDKN2A* group showed tumor growth inhibition and radiosensitization (Figure 5E). The increased radiosensitivity was most marked with the 4 Gy radiation dose, so this dose was selected as the radiation dose for subsequent experiments. *CDKN2A* knockdown led to a decrease in tumor cloning ability (Figure 5F). The clone formation rate following combined irradiation was markedly diminished in the sh*CDKN2A* group versus the control group. The decrease in *CDKN2A* expression was associated with decreased wound healing and invasion ability (Figures 5G,H). *CDKN2A* knockdown further inhibited the wound healing and invasion ability of irradiated cells. These findings indicate that *CKDN2A* knockdown suppresses CRC cell proliferation, invasion, and migration and enhances the radiosensitivity of CRC cells, supporting a contributory role of *CDKN2A* in radiation resistance. It should be noted that the correlation between *CDKN2A* and radiotherapy sensitivity at the clinical level has only been validated in the rectal cancer cohort, and should be treated with caution when extended to colon cancer.

Then, we constructed a subcutaneous tumor model (Figure 6A). Lower limb tumors were irradiated, while upper limb tumors were not irradiated. The results showed that *CDKN2A* knockdown made the tumor grow slowly after irradiation (Figures 6B–D). Immunohistochemistry showed that compared with NC group, sh*CDKN2A* group had more necrosis after irradiation (*P* < 0.05, Figure 6E).

**FIGURE 6:**
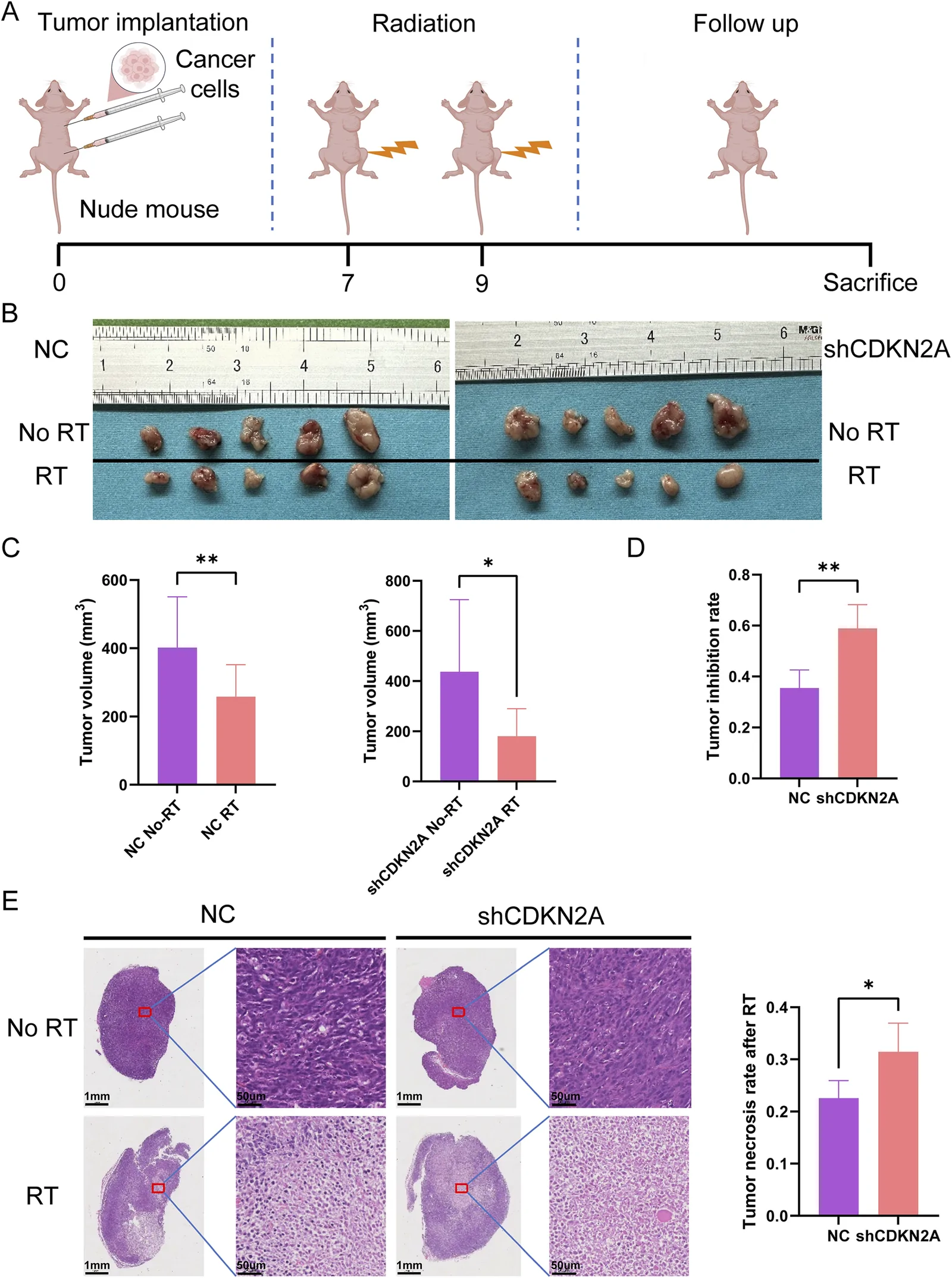
Animal experiment verified the relationship between CDKN2A and radiation sensitivity of colorectal cancer. **(A)** Construction and intervention of animal model. **(B)** Image, **(C)** volume of subcutaneous xenograft tumor, and **(D)** statistics of tumor growth inhibition rate. **(E)** Immunohistochemical image of tumor tissue and statistical bar chart of tumor necrosis rate. NC, negative control; No-RT, no-radiation; RT, radiation. *p < 0.05; **p < 0.01.

### Relationship between CDKN2A expression and prognosis

The link between *CDKN2A* expression and CRC prognosis was examined while considering multiple factors. Patients exhibiting high CDKN2A expression demonstrated inferior OS (Figure 7A), progress-free survival (Figure 7B), recurrence-free survival (Figure 7C), and disease-free survival (Figure 7D). We then analyzed the link between *CDKN2A* expression and clinicopathological features. *CDKN2A* expression was associated with the age ≤65 years (*P* = 0.002), lymph node metastasis (*P* = 0.03), distant metastasis (*P* = 0.014), and high TNM stage (*P* = 0.0051) (Figures 7E–H).

**FIGURE 7:**
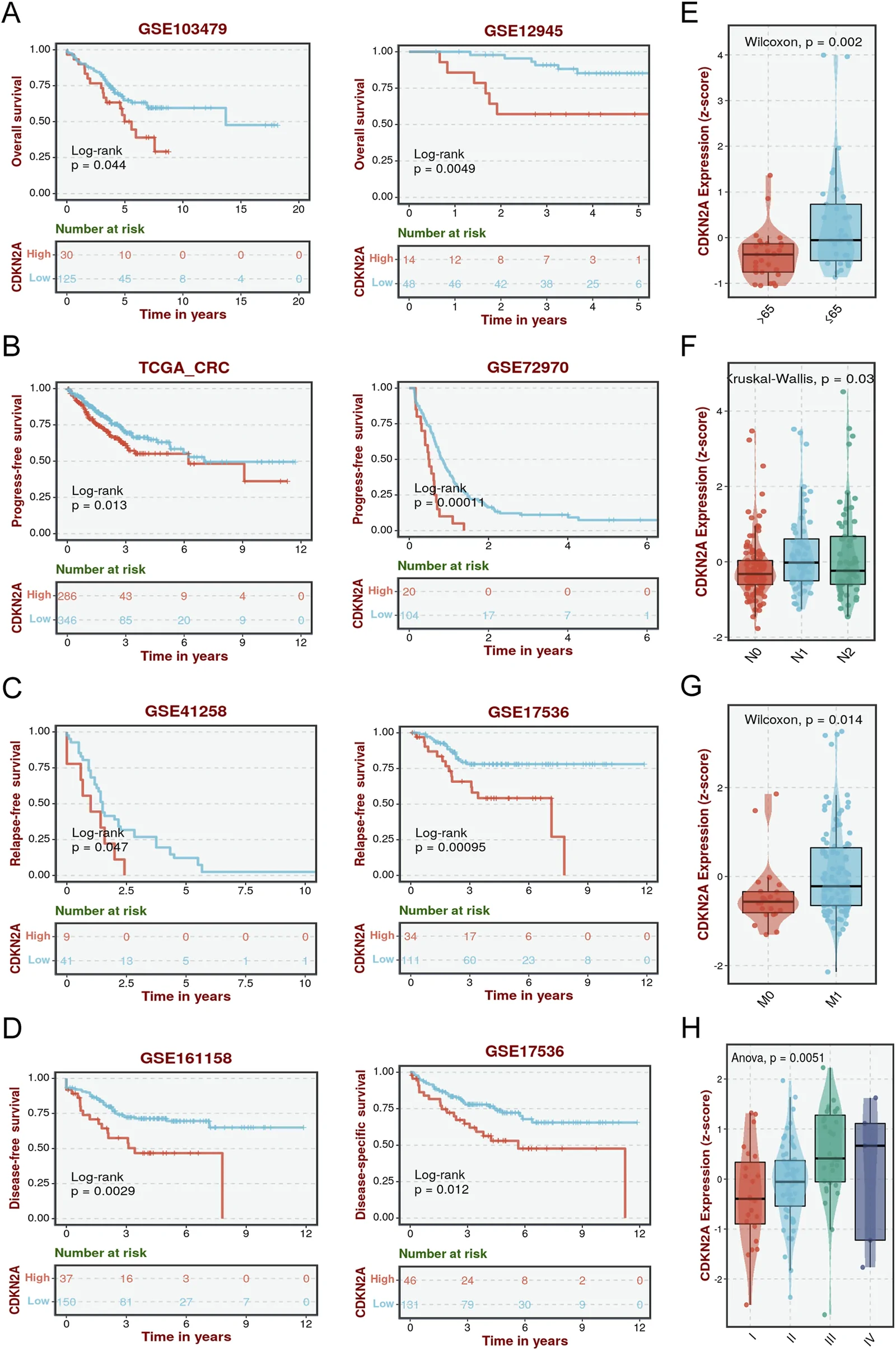
Relationship between CDKN2A expression and clinicopathological features. Kaplan–Meier curves plot of **(A)** overall survival, **(B)** progress-free survival, **(C)** recurrence-free survival, and **(D)** disease-free survival for patients with different CDKN2A expression levels. The box plots of the expression levels of CDKN2A in patients of different **(E)** ages, **(F)** N stages, **(G)** M stages, and **(H)** TNM stages.

### Relationship between CDKN2A expression and the tumor immune microenvironment

The relationship between the infiltration level of various immune cell types and *CDKN2A* gene expression was analyzed using bulk transcriptomics. The infiltration levels of M2-type macrophages, monocytes, and Tregs were positively linked to *CDKN2A* expression levels, whereas that of CD8^+^ T-cells was negatively linked to *CDKN2A* expression (Figure 8A; Supplementary Figure S3). Further analysis based on single-cell transcriptomics suggested that C6 and C9 tumor cell subtypes with high *CDKN2A* expression had weak interaction with CD8^+^ T-cells, but strong interaction with fibroblasts and endothelial cells (Figures 8B–D; Supplementary Figure S4).

**FIGURE 8:**
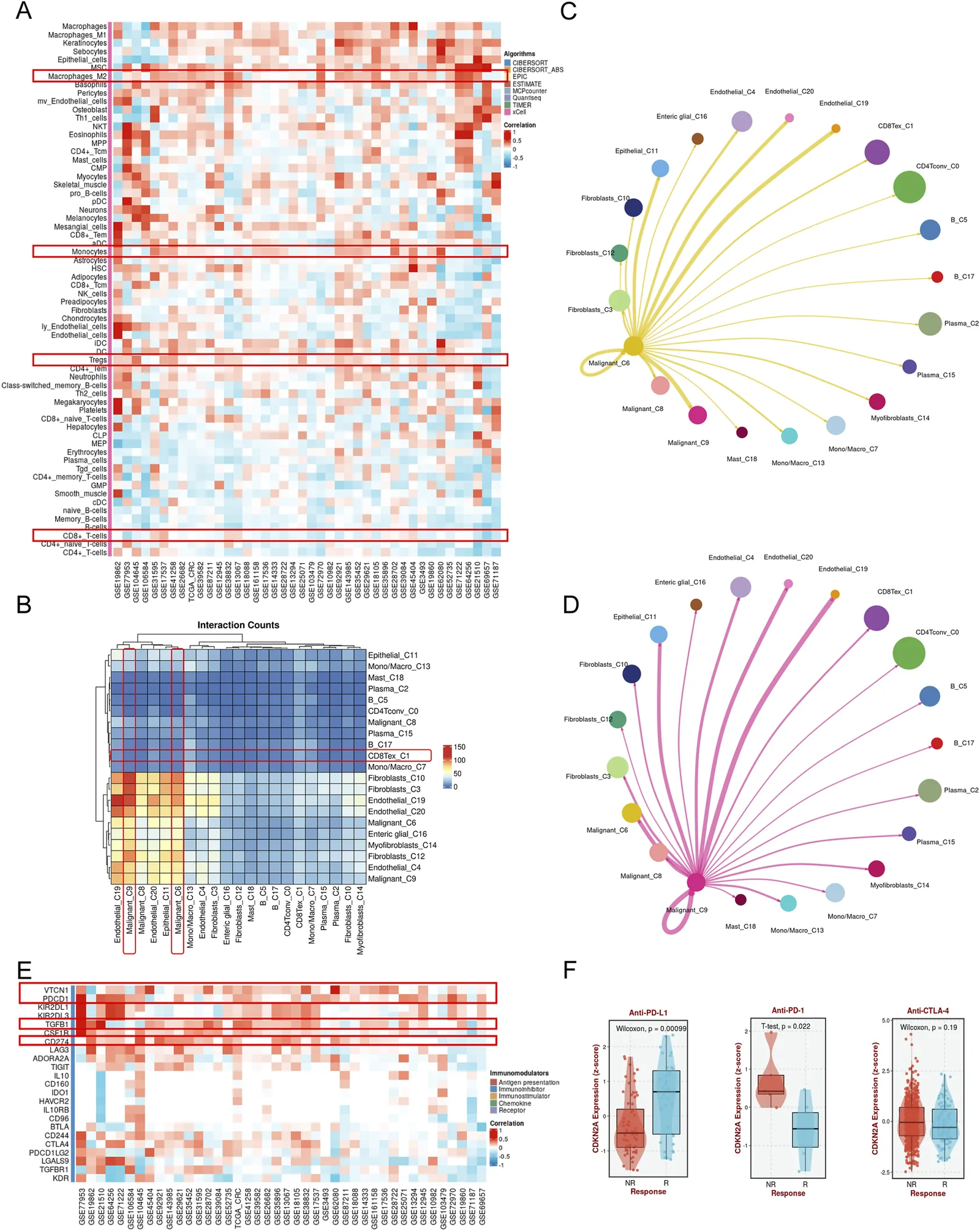
Relationship between CDKN2A expression and the tumor immune microenvironment. Heatmap of the correlation between the expression level of CDKN2A and the level of immune cell infiltration, as revealed by **(A)** bulk transcriptome and **(B)** single-cell transcriptome. The redder the color, the higher the correlation; the bluer the color, the lower the correlation. Circular graphs of the intensity of interaction between **(C)** C6 malignant cells or **(D)** C9 malignant cells and other types of cells. The thicker the lines between cells, the stronger the interaction between the cells. **(E)** Heatmap of the correlation between the expression level of CDKN2A and the level of immunomodulators. **(F)** Box plot of CDKN2A expression in different treatment response groups of patients after receiving immunotherapy. NR, no-response; R, response.

We then analyzed the molecular level in tumor tissue. The levels of immune inhibitors, such as VTCN1, TGFB1, PDCD1, and CD274, were positively linked to *CDKN2A* expression (Figure 8E; Supplementary Figure S5). In addition, the levels of expression of cytokine ligand and receptor such as CCL17, CXCL16 and CXCR5 were positively correlated with *CDKN2A* expression.

Further, the analysis of the effectiveness of immunotherapy showed that patients exhibiting elevated CDKN2A expression levels showed greater likelihood of responding to PD-L1 therapy (*P* < 0.001; Figure 8F; Supplementary Figure S4), yet demonstrated diminished likelihood of responding to anti-PD-1 therapy (*P* = 0.022), whereas response to CTLA-4 was not related to the expression level of *CDKN2A* (*P* = 0.19).

### Mechanism by which CKDN2A affects treatment sensitivity

The GO results suggested that the function of CKDN2A is related to biological processes such as intermediate filament cytoskeleton organization, regulation of macromolecule biosynthetic process, and DNA-templated regulation of transcription; and to molecular functions such as MHC class I receptor activity and N-methyltransferase activity (Figure 9A). The KEGG analysis demonstrated that *CDKN2A* expression links to transcription regulation, amino acid metabolism, lipid metabolism, and the vascular endothelial growth factor (VEGF)-signaling pathway (Figure 9B).

**FIGURE 9:**
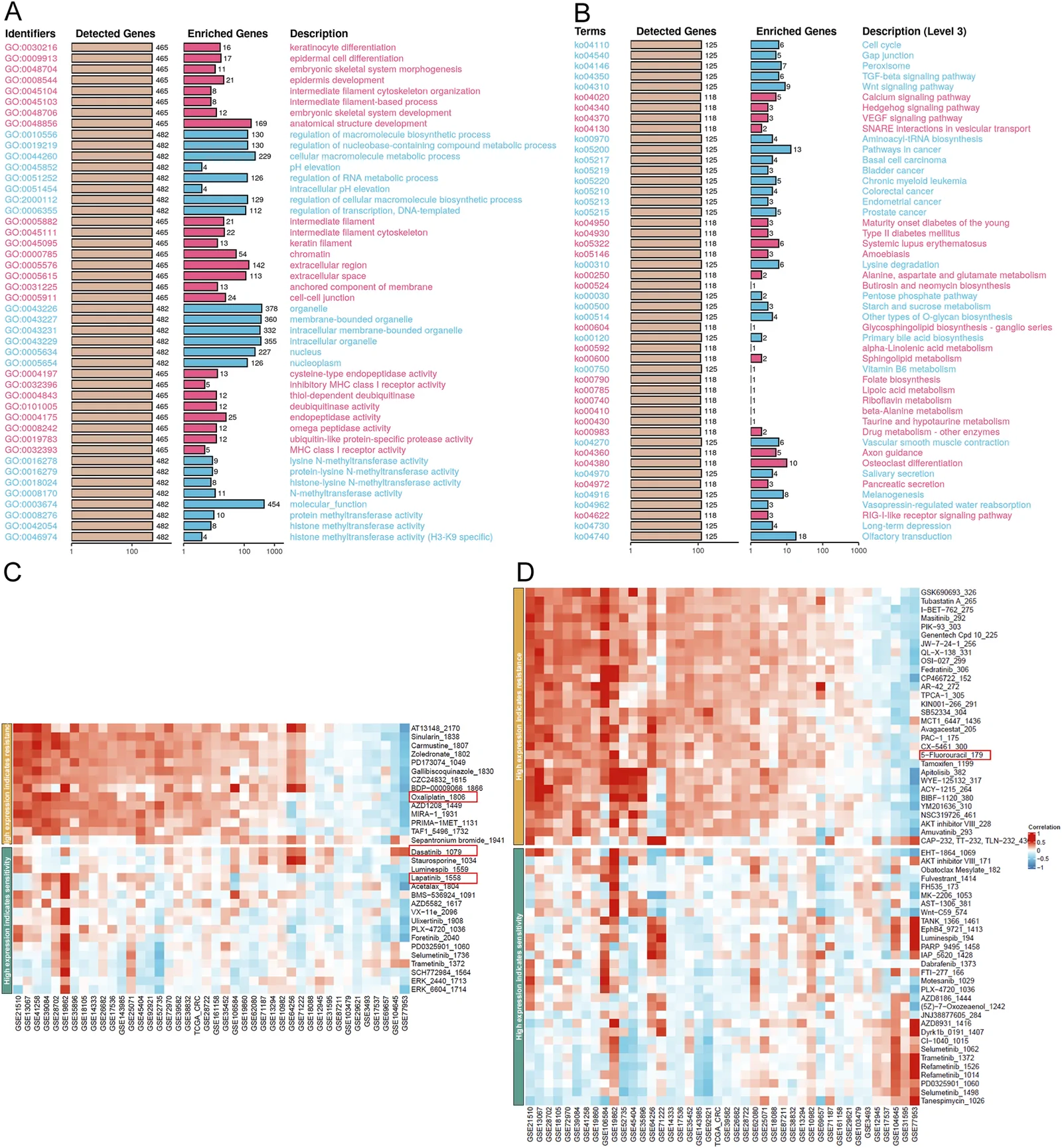
Mechanism exploration and drug sensitivity analysis. **(A)** GO enrichment and **(B)** KEGG pathway analysis based on the differentially expressed genes between the high and low CDKN2A expression groups. Heatmap of the relationship between the expression level of CDKN2A and the sensitivity to drug treatment derived from **(C)** GDSC2 and **(D)** GDSC1 databases. For the upper panel, redder color indicates stronger resistance to drug therapy in patients with high CDKN2A expression; for the lower panel, redder color indicates greater sensitivity to drug therapy in patients with high CDKN2A expression. GO, Gene Ontology; KEGG, Kyoto Encyclopedia of Genes and Genomes; GDSC, Genomics of Drug Sensitivity in Cancer.

Finally, we conducted a drug sensitivity analysis to screen for potentially beneficial drugs. The results suggested that the tumors of patients with high *CDKN2A* expression are more likely to be resistant to oxaliplatin and 5-FU (Figures 9C,D), may be sensitive to dasatinib, lapatinib, and GDC-0879 (Figure 9C; Supplementary Figure S6).

## Discussion

Through the analysis of bulk transcriptome, single-cell transcriptome, immunohistochemistry, and clinical information, this study identified the hub gene, *CDKN2A*, as playing a key role in CRC progression. This investigation demonstrated that elevated *CDKN2A* expression links to unfavorable prognosis and an immunosuppressive microenvironment in CRC patients. In addition, *in vitro* and *in vivo* experiments confirmed that *CDKN2A* knockdown reduces the proliferation, invasion, and migration abilities of tumor cells and increases their sensitivity to X-ray radiation. Collectively, these results suggest that the combination of radiation therapy and targeted intervention of *CDKN2A* has the potential to improve the prognosis of CRC.

This study demonstrated that *CDKN2A* gene expression exhibits marked elevation in CRC tissues, with elevated *CDKN2A* expression being closely linked to tumor resistance to radiotherapy and chemotherapy alongside unfavorable prognosis. *CDKN2A* serves a crucial function in cell cycle regulation. Its inactivation or mutation has been documented to correlate with disease advancement in various malignant tumors, including bladder cancer, pancreatic cancer, and hepatocellular carcinoma (Berggren et al., 2003; Raspollini et al., 2006; Heilmann et al., 2014; Tannapfel et al., 2001). Nevertheless, investigations regarding *CDKN2A*’s role in CRC remain relatively scarce. Hypermethylation of the *CDKN2A* gene has been documented as a predictor of unfavorable prognosis in CRC patients (Maeda et al., 2003; Bihl et al., 2012). While this study revealed that *CDKN2A* demonstrates high expression in CRC, its mutation frequency remains very low (approximately 1%). The findings align with previous studies demonstrating that elevated *CDKN2A* expression serves as an independent predictor of unfavorable prognosis in CRC patients (Shi et al., 2022; Cheng et al., 2024). Additional analysis of clinicopathological characteristics revealed that elevated *CDKN2A* expression correlated with multiple invasive features, including age ≤65 years, along with lymph node metastasis presence, distant metastasis, and advanced TNM stage. These findings indicate that *CDKN2A* participates in CRC malignant progression and holds value as both a prognostic biomarker and potential therapeutic target.

Tumor-infiltrating immune cells influence disease progression. The results of this study suggest that *CDKN2A* may regulate immune infiltration in CRC, based on the finding that *CDKN2A* expression was positively linked to the infiltration levels of tumor fibroblasts, M2-type macrophages, and monocytes, and negatively linked to CD8^+^ T-cell infiltration. M2-type macrophages and tumor-associated fibroblasts inhibit immune responses and promote tumor progression (Hu et al., 2025; Zhang et al., 2019). In tumors, monocytes can differentiate into immunosuppressive myeloid cells, further facilitating immune escape (Mortezaee, 2021); whereas reduced infiltration and inhibited functioning of CD8^+^ T-cells, the core effector cells of antitumor immunity, leads to weakened immune defense and immune surveillance functions (Ichiki et al., 2025; Spitzer et al., 2017). In addition, *CDKN2A* may affect the tumor immune microenvironment through cytokines such as IL-4 and IL-10 (Zhao et al., 2013; Zhu et al., 2021). Both our research and the study by (Dong et al., 2023) revealed that the *CDKN2A* expression level was positively linked to the levels of tumor immunosuppressive factors such as PDCD1 and CD274. Therefore, high *CDKN2A* expression may transform the tumor immune microenvironment into an inhibitory state, thus promoting the immune escape and tumor progression.

From the prediction results of TCIA database, we revealed that patients exhibiting elevated *CDKN2A* expression demonstrated superior responses to anti-PD-L1 treatment, while patients displaying diminished *CDKN2A* expression showed greater likelihood of responding to anti-PD-1 treatment. We speculate that the reason for these findings might be that in tumor microenvironments with high *CDKN2A* expression, the CD8^+^ T-cell infiltration is low, and the highly activated PD-1/PD-L1 pathway further leads to CD8^+^ T-cell depletion. Anti-PD-1 treatment does not reverse CD8^+^ T-cell depletion, whereas treatment with PD-L1 inhibitors may reverse the immunosuppressive tumor microenvironment. Additionally, when using PD-L1 inhibitors, PD-L2 can be retained to combine with repulsive guidance molecule B (RGMb), thereby promoting T-cell expansion (Xiao et al., 2014). In patients with low *CDKN2A* expression, the antitumor immune microenvironment is relatively favorable, and the level of CD8^+^ T-cell infiltration is high. At this time, the tumor-killing effect of CD8^+^ T-cells can be continuously activated through PD-1 inhibitors. Therefore, after further obtaining real-world data support, *CDKN2A* may be used as biomarker to guide the clinical selection of immunotherapeutic drugs.

The p16 protein encoded by *CDKN2A* functions to inhibit CDK4/6, which prevents Rb protein phosphorylation, thereby causing G1 phase arrest (Parkinson et al., 1997). Radiotherapy mainly acts on cells in the G2/M phases undergoing mitosis (Fan et al., 2020). Cancer cells with high p16 expression can undergo G1 phase arrest if subjected to stress, thereby reducing the damage to DNA in adverse environments and providing time for the repair of damaged DNA. This mechanism might explain why patients with CRC with high *CDKN2A* expression are insensitive to radiotherapy. 5-FU and oxaliplatin are representative drugs used for chemotherapy in CRC. However, this study found that CRC tumors with high *CDKN2A* expression are resistant to 5-FU and oxaliplatin. 5-FU is an S-phase-specific uracil analog (Onar et al., 2023), whereas the G1 phase arrest caused by *CDKN2A* makes 5-FU ineffective. Additionally, (Chen et al., 2024) reported that *CDKN2A* overexpression can activate the TP53-PI3K/Akt signaling pathway and induce resistance to 5-FU. The resistance to oxaliplatin may be related to cell cycle arrest and DNA damage repair. Therefore, targeting *CDKN2A* could reduce the radiochemotherapy resistance of CRC and become an important component of tumor treatment.

This investigation systematically explored the expression pattern, clinical significance, and regulatory function of *CDKN2A* in CRC regarding its effect on response to radiotherapy and its effects on the tumor immune microenvironment. It provides evidence supporting *CDKN2A* as a potential therapeutic target and prognostic biomarker. However, this investigation has some limitations: Firstly, the main conclusions are based on retrospective analysis of public databases. Although some research results were verified through *in vitro* experiments, additional prospective clinical studies, gain-of-function and rescue experiments in CRC cell lines with low endogenous *CDKN2A* expression are required to confirm the biological function of *CDKN2A* and the clinical applicability of these findings. The use of 1 cell line also limits the generalizability of findings. Secondly, although *in vivo* experiments have also found that *CDKN2A* affects radiosensitivity, the use of dual-tumor animal models may lead to mutual influences between the two tumors through cytokines and other mechanisms. Therefore, the findings from animal experiments should be interpreted with caution. Thirdly, the molecular mechanisms by which *CDKN2A* affects radiotherapy sensitivity and immune microenvironment have not been fully elucidated. The TGF-β pathway was identified by functional enrichment and remains a bioinformatic prediction without experimental validation. Moreover, the assertion that CRC tumors with high *CDKN2A* expression may respond to agents such as dasatinib and lapatinib is based on algorithm predictions, and their actual efficacy still needs to be verified through conducting clinical studies.

## Conclusion

In summary, this study confirmed that *CDKN2A* is overexpressed in CRC, and that high *CDKN2A* expression is closely related to poor response to radiotherapy and chemotherapy, poor prognosis, and an immunosuppressive microenvironment. Targeted inhibition of *CDKN2A* enhances the sensitivity of tumor cells to radiotherapy and may modulate the immunosuppressive state, potentially providing a new strategy for improving the effectiveness of treatment in patients with CRC.

## Data Availability

The original contributions presented in the study are included in the article/Supplementary Material, further inquiries can be directed to the corresponding authors.
