# Supplementary material for: Association of CDKN2A overexpression with poor prognosis, radioresistance, and immunosuppression in colorectal cancer
Source: Front Cell Dev Biol. 2026 Oct 16;14:1955910. doi: 10.3389/fcell.2026.1955910 (PMC13719471; doi:10.3389/fcell.2026.1955910)
Supplement: Supplementary file 1 [file Supplementaryfile1.docx]

**Supplementary methods**

**R****everse-transcription PCR**

RNA extraction from experimental samples was conducted utilizing the TRIzol extraction protocol. Subsequently, first-strand cDNA synthesis was carried out employing a commercial reverse-transcription kit (Yeasen Biotechnology Co. Ltd., Shanghai, China) following these thermal cycling parameters: initial incubation for 2 min at 42°C, proceeded by 5 min at 25°C, a 30-min extension phase at 42°C, enzyme inactivation step at 85°C for 5 min, and a concluding 10 min hold at 4°C. Total RNA served as the initial template for this reaction. The reverse-transcription PCR (RT-PCR) procedure was executed using 2 × Rapid Taq Master Mix (Vazyme, Nanjing, China) following these thermal cycling parameters: preliminary denaturation at 95°C for 3 min, succeeded by 35 cycles consisting of 95°C for 15 s, 60°C for 15 s, and 72°C for 15 s per kilobase. The cycle threshold values were recorded for each reaction well.

**Western blot**

A protease inhibitor was incorporated into the radioimmunoprecipitation assay (RIPA) buffer. Protein quantification was performed using the BCA protein assay kit, after which 15% sodium dodecyl-sulfate polyacrylamide gel electrophoresis (SDS-PAGE) (UE Medical, China) was conducted. Following electrophoresis, proteins were transferred onto a polyvinylidene difluoride (PVDF) transfer membrane, which was then blocked with 5% skimmed milk powder for 2 h at room temperature. Subsequently, the membrane was incubated overnight at 4℃ with primary antibodies (GAPDH 1:5000, CDKN2A 1:4000) (Proteintech, Rosemont, IL, USA). The PVDF membrane was further incubated with secondary antibody (HRP-conjugated goat anti-rabbit 1:4000) (Proteintech) for 1 h, and protein expression was visualized through chemiluminescence detection.

**Cell viability assessment**

Cell viability was assessed utilizing the Cell Counting Kit-8 (CCK-8) viability assay (MedChemExpress, Princeton, NJ, USA). In detail, 2 × 10^3^ cells were plated into separate wells of a 96-well plate and exposed to the specified treatments. Cell viability was determined following 24 h. Then, 10 μL of CCK-8 solution was introduced to each well, followed by incubation at 37℃ for 45 mins to 2 h. Absorbance at 450 nm was detected using a microplate reader.

**Wound-healing and transwell assays**

Cells were placed into 6-well plates at 1 × 10⁶ cells per well and maintained at 37°C until reaching 90% confluency. A standardized scratch was generated across each well using a 200-μL pipette tip. Detached cellular debris was eliminated through phosphate-buffered saline washing, followed by cell irradiation. Wound healing progression was monitored at 0- and 24-hours post-scratch using an inverted microscope, with image acquisition performed at these designated time-points. Scratch area measurements were conducted and cell migration rates were determined using ImageJ software (v. 1.52i).

After irradiation, cells underwent dilution in serum-free medium before being placed into the upper chamber. Medium containing 10% FBS was added to the lower chamber as a chemoattractant. After 48 h of incubation, cells that failed to invade were eliminated from the upper membrane surface. Cells that had migrated to the lower surface underwent fixation with 4% formaldehyde solution, crystal violet staining, and tap water rinsing. Quantification of invasive cells was performed using ImageJ software (v. 1.52i).

**Immunohistochemistry**

The paraffin was frozen and sliced. The thickness of the slice was 4–5 μM. After the slice was flattened in 43℃ water, the tissue was fished out with a slide, baked at 43℃ for 24 h, and dried at 60℃ for 1 h. Dewaxing was carried out according to the following procedures: xylene I for 10 mins, xylene II for 10 mins, anhydrous ethanol I for 5 mins, anhydrous ethanol II for 5 mins, 95% ethanol for 5 mins, 90% ethanol for 5 mins, 80% ethanol for 5 mins, 70% ethanol for 5 mins, ultrapure water for 5 mins, ultrapure water for 5 mins. Dyeing was carried out according to the following procedures: hematoxylin for 5 mins, washing until no dye flows out, PBS 5 mins, eosin for 1 min, 95% ethanol for 3 s, anhydrous ethanol for 2 s, washing until no dye flows out, anhydrous ethanol for 10 mins, xylene for 10 mins. Use a neutral gum seal.

**Supplementary figures**

**Supplementary Figure 1.** The expression of CDKN2A in colorectal cancer. UMAP of the distribution of (A) 21 cell clusters and (B) 12 cell types identified from single-cell transcriptomic dataset. The proportion of 12 cell types in the (C) total population and (D) each patient. The expression of CDKN2A in (E) 21 cell clusters and in (F) 12 types of cells. (G) Immunohistochemical detection of the expression of CDKN2A in tumor tissues and normal tissues of colorectal cancer.

**Supplementary Figure 2.** Full uncropped gels and blots images. The position of (A) CDKN2A and (B) GADPH in full uncropped gels. (C) The western blot showing the protein expression levels of CDKN2A in different intervention groups. (D) The protein content is calculated as the relative value based on the color intensity of the protein bands in the gel.

**Supplementary Figure 3.** Relationship between CDKN2A expression and the tumor immune cells infiltration. Heatmap of the correlation between the expression level of CDKN2A and the level of immune cell infiltration as revealed by bulk transcriptome.

**Supplementary Figure 4.** The intensity of interaction between tumor and other cells and its response to immunotherapy. Circular graphs of the intensity of interaction between (A) C6, (B) C8, or (C) C9 malignant cells and other types of cells. The thicker the lines between cells, the stronger the interaction between the cells. Box plot of CDKN2A expression in different treatment response groups of patients after receiving (D) anti-PD-L1, (E) anti-PD-1, or (F) anti-CTLA-4 treatments. Using AUC curve to predict the curative effect of immunotherapy. NR, no-response; R, response; AUC, area under the curve.

**Supplementary Figure 5.** Heatmap of the correlation between the expression level of CDKN2A and the level of immunomodulators.

**Supplementary Figure 6.** Heatmap of the relationship between the expression level of CDKN2A and the sensitivity to drug treatment. Heatmap of the relationship between the expression level of CDKN2A and the sensitivity to drug treatment derived from (A) GDSC2, (B) GDSC1, (C) CTRP, and (D) PRISM databases. For the upper panel, redder color indicates stronger resistance to drug therapy in patients with high CDKN2A expression; for the lower panel, redder color indicates greater sensitivity to drug therapy in patients with high CDKN2A expression. GDSC, Genomics of Drug Sensitivity in Cancer; CTRP, Cancer Therapeutics Response Portal; PRISM, Profiling Relative Inhibition Simultaneously in Mixtures.
